# Atlanta Society of Medicine

**Published:** 1889-07

**Authors:** 


### THE ATLANTA SOCIETY OF MEDICINE.

MEETING, June 24th.

President Dr. Hunter P. Cooper in the chair.

Dr. W. S. Elkin reported an interesting case of successful removal of foreign body from the bladder.

The patient was a young man, nineteen years of age, addicted to the habit of masturbating, who frequently introduced bull-rushes into his urethra during the act. On this occasion the specimen shown, seventeen and one-half inches in length, slipped from his grasp and glided back into his bladder. It remained in bladder for several hours, producing great irritation and frequent micturition. Removal was effected by means of a lithotrite, the patient making a good recovery.

Drs. Nicholson and Cooper reported cases of foreign bodies in bladder which had come under their observation.

Dr. J. W. Duncan mentioned an unusual accident which happened to a brother physician in the city recently. The doctor introduced a soft catheter for his patient, and on removal found that two or three inches of the catheter had remained in the bladder. The doctor, nothing daunted, assured his patient tha he need have no uneasiness as the catheter would soon dissolve. Fortunately the patient voided the piece entire in a few days after.

Dr. Nicholson reported a case of fracture of both bones of the leg in a patient eighty-six years old. The fibula was fractured in two places and the tibia was fractured and dislocated, protruding through the skin three or four inches. The injury was the result of jumping from a moving train. The most peculiar features of the case were the absence of shock and apparent complete anæsthesia of limb, the patient suffering almost *nil* during the reduction of the fracture and dislocation. The injury was dressed antiseptically and a plaster of paris splint applied.

Drs. Cooper and Bizzell reported similar cases seen in hospitals.

Dr. McRae mentioned a case of compound comminuted fracture of both bones of the leg, which healed beautifully under antiseptic dressings and plaster splints.

Dr. Hardon reported a case of removal of hydatid mole on account of obstinate vomiting. Also a case of Battey's operation where one of the ovaries was found partially ossified.

Dr. McRae reported a case of amenorrhœa of three and one-half years' standing, cured by electricity, emenagogues and massage. The patient, a young lady twenty-one years old, began menstruating at the age of fourteen, and menstruated regularly until nearly eighteen, when, from exposure and catching cold, the menses disappeared, not returning until the last few months. The patient lost flesh gradually, falling in weight from one hundred and sixty pounds to less than one hundred pounds, and passed through the hands of a number of physicians and quacks. The faradic battery was used. One electrode was introduced into the uterus, and the other alternately over the spine and hypogastrium.

Dr. Hardon mentioned several cases of amenorrhœa cured by faradism alone.

Dr. Jones reported a case of hour-glass contraction of the uterus.

Dr. Hardon said he had never seen a case of hour-glass contraction where ergot had not been administered, and advised against its use in obstetric practice.

Dr. Cooper said he thought ergot was unreliable and that it should be abolished.

Dr. Bizzell considers ergot a valuable drug, especially in post-partum and other hemorrhages.

Dr. Crawford mentioned two cases of hour-glass contractions, the result of too early attempts at the removal of the placenta.

